# Through the Narrative Looking Glass: Commentary on “Impact of Electronic Health Records on Information Practices in Mental Health Contexts: Scoping Review”

**DOI:** 10.2196/38513

**Published:** 2022-05-04

**Authors:** Charlene Weir

**Affiliations:** 1 Department of Biomedical Informatics University of Utah Salt Lake City, UT United States

**Keywords:** electronic health records, psychiatry, mental health, electronic medical records, health informatics, mental illness, scoping review, clinical decision support

## Abstract

The authors of “Impact of Electronic Health Records on Information Practices in Mental Health Contexts: Scoping Review” have effectively brought to our attention the failure of the electronic health record (EHR) to represent the human context. Because mental health or behavioral disorders (and functional status in general) emerge from an interaction between the individual’s characteristics and the social context, it is essentially a failure to represent the human context. The assessment and treatment of these disorders must reflect how the person lives, their degree of social connectedness, their personal motivation, and their cultural background. This type of information is best communicated both through narrative and in collaboration with other providers and the patient—largely because human social memory is organized around situation models and natural episodes. Neither functionality is currently available in most EHRs. Narrative communication is effective for several reasons: (1) it supports the communication of goals between providers; (2) it allows the author to express their belief in others’ perspectives (theory of mind), for example, those who will be reading these notes; and (3) it supports the incorporation of the patient’s personal perspective. The failure of the EHR to support mental health information data and information practices is, therefore, essentially a failure to support the basic communication functions necessary for the narrative. The authors have rightly noted the problems of the EHR in this domain, but perhaps they did not completely link the problems to the lack of functionality to support narrative communication. Suggestions for adding design elements are discussed.

## Introduction

Through their scoping review of mental health data and the electronic health record (EHR), the authors of “Impact of Electronic Health Records on Information Practices in Mental Health Contexts: Scoping Review” have brought to center stage the failure of the EHR to represent the human context [1]. Mental health or behavioral disorders (and functional status in general) emerge from an *interaction* between the individual’s characteristics and the social context. As a result, the assessment and treatment of these disorders must reflect how the person lives, their degree of social connectedness, their personal motivation, and their cultural background—in other words: the human context. This failure of the EHR to support both information data (eg, missing or “fuzzy” data) and information practices (processes) for mental health information is a feature, not a bug. Specifically, EHRs have systematically avoided supporting text data—partially because electronic text is seen as hard to use [2] and due to the belief that structured data is more accurate. However, it is not just that providers *prefer* to tell the patient’s story in narrative rather than structured data forms [3] or that mental health data is “soft” data, it is that it is much too difficult to get a sense or gist of the patient’s situation through structured data and is much less cognitively efficient. In other words, accuracy is in the eye of the beholder. Some studies have noted the narrative is more accurate for mental health data, even if different text is used as descriptors [4]. One reason for the power of the narrative is that memory is organized around situation models and episodic mental representations, which are best communicated in story form [5]. Humans can grasp a situation much more rapidly through a story than through a list of facts [5]. Putting together “pieces” of data to get a gist of the patient’s situation is significant work, whereas distilled information has better comprehension and is associated with better decision-making [6]. Narrative communication is effective for several reasons: (1) it supports the communication of goals between providers; (2) it allows for the author to express their belief in others’ perspectives (theory of mind), for example, those who will be reading these notes; and (3) it supports the incorporation of the patient’s personal perspective.

## Goals

Documenting and tracking clinical goals is at the heart of care processes and communication in general [7]. The clinical goals for mental health patients almost always involve some aspect of context (which, in turn, requires specific descriptions of that context). The question “is the therapeutic treatment working?” requires data about the patient’s work situation, personal relationships, or the patient’s motivation [8].

## Communication

Communicating mental health information to other providers is complex because people of many different roles care for these patients compared to patients with other disorders. Documentation must then be “tailored” to the audience and to the perspectives of differing roles (theory of mind), which requires significant amounts of working memory [9].

## Patient Preferences

The patient’s preferences are often idiosyncratic, embedded in the social context, and specific to location and time. The EHR is a limited representation of patient preference data. A story about the patient’s wishes is generally the most effective way of communicating preferences and planning care [10].

## Conclusion

The failure of the EHR to support mental health information data and information practices is, therefore, really a failure to support the basic communication functions necessary for the narrative. The authors have rightly noted the problems of the EHR in this domain, but perhaps they did not completely link the problems to the lack of functionality to support narrative communication. Links to the clinical goals of other clinicians, a specific location for the patient’s story, temporal links to clinical episodes, and the ability to annotate the clinical notes of others in order to understand one’s impressions would help communicate the patient’s story. Improving the use of natural language processing and building ontologies of context would also help. Additionally, addressing these functions would also address several of the issues raised in the review, specifically, missing data, sensitive data, and collaborative decision-making information. Future work in the arena of EHRs could create tools and spaces for narrative, patient preferences, collaborative discourse, and shared collaborative documentation [11].

## Abbreviations

EHR: electronic health record
